# Disruption of rhythms of molecular clocks in primary synovial fibroblasts of patients with osteoarthritis and rheumatoid arthritis, role of IL-1β/TNF

**DOI:** 10.1186/ar3852

**Published:** 2012-05-23

**Authors:** Stefanie Haas, Rainer H Straub

**Affiliations:** 1 Laboratory of Experimental Rheumatology and Neuroendocrino-Immunology, Division of Rheumatology, Department of Internal Medicine I, University Hospital, F. J. Strauss-Allee 11, Regensburg, 93053, Germany

## Abstract

**Introduction:**

Circadian rhythms play an important role in the body and in single cells. Rhythms of molecular clocks have not been investigated in synovial fibroblasts (SF) of patients with osteoarthritis (OA) and rheumatoid arthritis (RA). The study was initiated to fill this gap and to study effects of interleukin (IL)-1β/tumor necrosis factor (TNF) on rhythmicity in synovial fibroblasts of RA and OA patients.

**Methods:**

The presence of BMAL-1, CLOCK, Period 1 and Period 2 proteins in synovial tissue was investigated by immunofluorescence. The presence of mRNA of molecular clocks was studied during 72 h by qPCR. Characteristics of rhythms were studied with time series analysis.

**Results:**

BMAL-1, CLOCK, Period 1 and Period 2 proteins were abundantly present in synovial tissue of OA, RA and controls. Receiving synovial tissue at different operation time points during the day (8:00 am to 4:00 pm) did not reveal a rhythm of BMAL-1 or Period 1 protein. In OASF and RASF, no typical rhythm curve of molecular clock mRNA was observed. Time series analysis identified a first peak between 2 and 18 hours after synchronization but a period was not detectable due to loss of rhythm. TNF inhibited mRNA of CLOCK, Period 1 and Period 2 in OASF, while IL-1β and TNF increased these factors in RASF. This was supported by dose-dependently increased levels in MH7A RA fibroblasts. In RASF, IL-1β and TNF shifted the first peak of BMAL-1 mRNA to later time points (8 h to 14 h).

**Conclusion:**

Rhythmicity is not present in primary OASF and RASF, which is unexpected because fibroblasts usually demonstrate perfect rhythms during several days. This might lead to uncoupling of important cellular pathways.

## Introduction

Symptoms, such as swelling, pain, stiffness, and functional ability, follow a circadian rhythm in patients with rheumatoid arthritis (RA) [1]. The circadian change of symptoms depends on increased levels of proinflammatory cytokines in the late night and early morning [1-4], which can be blocked by night time application of glucocorticoids [4-6]. Circadian changes on the level of the entire body might be translated into rhythmic phenomena in peripheral cells of inflamed tissue. While oscillations of the entire system are dependent on external synchronizers, such as light, and are called circadian rhythms, undulations of intracellular molecular clock networks are dubbed daily rhythms depending on synchronization with, for example, serum shock. These intracellular daily rhythms have never been investigated in primary material of patients with RA and osteoarthritis (OA).

The circadian rhythm is generated in the superordinate hypothalamic nucleus suprachiasmaticus [7], and this rhythm can be transferred to peripheral cells of the body by hormonal and neuronal signals [8-12]. Under physiological conditions, this was described for hepatocytes, cardiomyocytes, peripheral blood mononuclear cells, natural killer cells, CD4+ T cells and others [8-13]. After synchronization with serum shock *in vitro*, rhythms of molecular clocks are often self-sustained under constant culture conditions [12,14,15]. This has been demonstrated for neurons of the nucleus suprachiasmaticus [14], T cells [12] and fibroblasts [15]. Particularly, fibroblasts demonstrated a self-sustained rhythm with three regular periods and stable amplitudes over 72 hours [15]. This peripheral rhythm of fibroblasts can be modulated by systemic factors, such as glucocorticoids [16]. It is thought that these rhythms serve an overall coupling of important bodily functions which is, for example, reflected in coupling of feeding behavior and cardiomyocyte responsiveness to ingested fatty acids [8].

Coupling phenomena can be observed on the level of the entire body as a simultaneous up-regulation of cooperative hormones, such as cortisol and norepinephrine, which both rise during the early morning hours to support release of energy-rich substrates from adipose tissue and liver and both decrease at midnight kicking off nightly immune phenomena [17]. Hormones of the hypothalamic-pituitary adrenal (HPA) axis and the sympathetic nervous system (SNS) are coupled in healthy subjects but are uncoupled in patients with Crohn's disease, ulcerative colitis and systemic lupus erythematosus [18,19]. Circadian rhythms of cortisol can be altered in patients with RA [20,21]. We recently reported that coupling phenomena are important to suppress proinflammatory cytokines in primary synovial cells of patients with RA [22].

It can be hypothesized that coupling of molecular clocks that guide endogenous cellular rhythms on the peripheral cellular level play an important role in inflammation. Molecular clocks coordinate timely and functionally coupled and uncoupled cellular phenomena. Alterations of this coordination by deletion of important molecular clocks aggravate experimental arthritis as recently demonstrated for the first time in mice [23]. There is a regular cross-talk of molecular clocks and TNF in fibroblasts [23]. However, rhythms of molecular clocks have never been investigated in primary material of RA and OA patients.

This study was initiated to examine the presence of molecular clocks in primary tissue of OA and RA patients. We hypothesized that density of cells positive for molecular clocks can change during the day in these patients in a rhythmic fashion. We further hypothesized that typical daily rhythms of molecular clocks can be demonstrated in the primary fibroblast of OA and RA patients and that proinflammatory cytokines, such as IL-1β and TNF, influence the presence and rhythm of molecular clocks.

## Materials and methods

### Patients and control subjects

Synovial tissue was obtained from RA and OA patients during knee replacement surgery as described earlier [24]. Control synovial tissue samples were obtained from patients with joint trauma during routine arthroscopy or open joint surgery for diagnostic and therapeutic procedures. All patients were informed of the purpose of the study and gave written consent. The study was approved by the Ethics Committee of the University of Regensburg. Characteristics of patients are given in Table 1.

**Table 1:** Characteristics of patients

	Osteoarthritis	Rheumatoid arthritis
number	17	17
age (yr)	68.8 ± 1.6	62.1 ± 3.0
sex (f/m)	14/3	13/4
C-reactive protein (mg/l)	9.4 ± 4.1	40.1 ± 14.6*
erythrocyte sedimentation rate (mm)	12.4 ± 2.6	41.5 ± 9.2*
*medication*		
prednisolone (%)	N.A.	88.0
daily prednisolone (mg/d)	N.A.	4.3 ± 0.5
methotrexate (%)	N.A.	35.3
anti-TNF therapy (%)	N.A.	5.9
leflunomide (%)	N.A.	29.4
cyclosporin A (%)	N.A.	5.9
peripherally acting opioidergic drugs (%)	41.2	35.3
Non-steroidal antiinflammatory drugs (%)	70.6	64.7

*P < 0.05 for the comparison vs. osteoarthritis. Abbreviations: N.A., not applicable.

Data are given as means ± SEM, percentages in parentheses, and ranges in brackets

### Immunofluorescence of synovial tissue

The tissue preparation for histological studies was performed as previously described [24]. Cryosections (5 μm) of at least three different formaldehyde-fixed synovial tissue samples from each patient/control were used. We used primary antibodies against the clock proteins BMAL-1 (1:100, polyclonal, rabbit versus human, Dianova, Hamburg, Germany), CLOCK (1:100, polyclonal, rabbit versus human, Calbiochem, Darmstadt, Germany), Period 1 (1:100, polyclonal, rabbit versus human, Millipore, Eschborn, Germany), Period 2 (1:100, polyclonal, rabbit versus human, Biozol, Eching, Germany). The secondary antibody was coupled to the fluorescent dye Alexa Fluor 546 (polyclonal, goat versus rabbit, Invitrogen, Karlsruhe, Germany). Non-specific binding sites were blocked with phosphate-buffered saline containing 10% fetal bovine serum, 10% bovine serum albumin, and 10% normal chicken serum or 10% normal goat serum for 45 minutes at room temperature. The samples were then incubated with the respective primary antibody for 3 hours at room temperature, washed and then incubated with specific secondary antibody for 90 minutes. After 4'-6-diamidino-2-phenylindole (DAPI; Roche, Mannheim, Germany) staining, slides were covered with fluorescence mounting medium (DAKO, Hamburg, Germany) and stored at 4°C until microscopy (performed within four days). Control staining was carried out with the secondary antibody alone or using unspecific rabbit serum instead of the primary antibody. Control staining always yielded a negative result.

### Synovial fibroblasts

Mixed synoviocytes were isolated from synovial tissue as described before [24]. The cells were cultured over three to six passages yielding a homogenous cell population of synovial fibroblasts. In an earlier study [25], it was demonstrated that only after seven to eight passages more than 10% of the genes were differentially expressed. Thus, passage 6 was used as the upper limit. The same study also demonstrated that doubling rate was constant for up to five passages and decreased after passages 6 to 8 [25]. With this information in mind, we focused on passages 3 to 6, which was necessary due to the enormous amount of cells needed.

Preliminary experiments did not show a difference in molecular clock mRNA in synovial fibroblasts of passage 3 to 6. Per well, 150,000 cells were cultured in culture medium (RPMI 1640, Sigma, Steinheim, Germany) containing 10% fetal bovine serum, 1% penicillin/streptomycin and 0.1% amphotericin B for 24 hours. Then, cells were starved for 24 hours in medium without fetal bovine serum in order to synchronize cellular rhythms as previously reported [26]. To start daily clock rhythms, cells received a serum shock (10% fetal bovine serum) in culture medium described above. At time t = 0, cells were treated without additional cytokines (control) or with IL-1β or TNF. Starting at t = 0, cells were harvested every 2 hours for a period of 72 hours (this is a high time resolution in these types of experiments). Harvested cells were washed and stored in RNA later solution (Sigma, Steinheim, Germany) at 4°C and then at -30°C until RNA isolation.

### MH7A synovial fibroblast cell line

Some of the experiments were repeated with the MH7A synovial fibroblast cell line. This cell line was derived from immortalized synovial fibroblasts of a patient with RA using the SV40 T antigen [27]. MH7A were purchased from Riken, Japan, with informed consent of Central Research Laboratories, Kissei Pharmaceutical Co., Ltd., Nagano, Japan. Similar as mentioned for primary synovial fibroblasts, synchronization was established by serum shock. Cells were incubated at indicated concentrations of IL-1β and TNF. RNA isolation was performed as described.

### RNA isolation and quantitative PCR

Since most studies on molecular clocks studied mRNA and because protein determination with Western blot was extremely time-consuming (preliminary studies), we used quantitative PCR to detect levels of molecular clock mRNA. RNA was isolated according to the manufacturer's instructions (Nucleo Spin RNA II Kit, Macherey Nagel, Düren, Germany). cDNA was converted from total RNA (RevertAid First Strand cDNA Synthesis Kit; Fermentas, St. Leon-Roth, Germany). For quantitative PCR (qPCR), 1 μl of cDNA preparation, 1 μl of specific primer (100 pmol/μl; Eurofins MWG Operon, Ebersberg, Germany) and Brilliant II SYBR Green qPCR Master Mix (Agilent Technologies, Waldbronn, Germany) were applied in a total volume of 10 μl. The PCR reaction was evaluated by melting curve analysis according to the manufacturer's instructions (Thermal Cycler 7900 HAT, Applied Biosystems, Darmstadt, Germany). Each quantitative PCR was performed in triplicate.

The following primers were used: BMAL-1 forward ATCAGACGATGAATTGAAACAC, BMAL-1 reverse TCATTCTGGCTGTAGTTGAGGA; CLOCK forward ACCCTTCCTCAACACCAAC, CLOCK reverse GACTGGGAATTTATGGACTGAC; Period 1 forward AGTTCCATTGCCTACAGCC, Period 1 reverse GAAGTGCTGTCATGAGTTCC; Period 2 forward GCATTTCATTAAGTGCGTCC, Period 2 reverse GCTTCTCTCTGTCCTCCTTC; Per3 forward GTTGTCGCCATCGTTTTTGCC, Per3 reverse GCTTTGTGCCTCCCACTTTTCC; Cryptochrome 1 forward GGATTGATGCCATCATGACAC, Cryptochrome 1 reverse CCTTCATTCCTTCTTCCCAAC; Cryptochrome 2 forward GCGCTGCGTTTACATTCTC, Cryptochrome 2 reverse CTTGTGTCCAAATCTTCCAGAG; Rev-erbα forward CCCTTCTTCCTCATCTTCCTC, Rev-erbα reverse GATGTTGCTGGTGCTCTTG.

Furthermore, Cdc2 was detected in order to estimate the interrelation to the cell cycle because this factor is detrimental for cell cycle progression [28]. The following primers were used for Cdc2: forward ACTGGCTGATTTTGGCCTTGC, reverse AGTTGAGTAACGAGCTGACCCC. The housekeeper 18s was used as control using the following primers: forward CGGCTACCACATCCAAGGAA, reverse GCTGGAATTACCGCGGCTGC. Each gene was normalized to the house keeper mRNA of 18s. The ratio of the mRNA of a specific gene divided by the mRNA of 18s at t = 0 was defined as 1.00 and ratios of every following time point were calculated in relation to t = 0.

### Statistical analyses

Medians of groups were compared by Mann-Whitney U test and Box Plots were demonstrated to respect the non-normal distribution of data (Sigma Plot 11.0, Systat Software, Inc., Erkrath, Germany). For time series analysis, cubic regression was used for the interrelation of time of day and density of cells positive for molecular clocks in synovial tissue (Sigma Plot). In addition, autocorrelation analyses were used to identify a possible period of the rhythm (PASW Statistics, 18.0, IBM SPSS via IBM Germany, Ehningen, Germany). We did not use a sine or cosine wave fitting model because it was unclear whether or not oscillations really exist. Trigonometric wave fitting always yields a rhythm and, thus, it is not useful to apply this technique in a situation where the presence of oscillation is not really known. Elapsed time between serum shock synchronization and first peak of molecular clock mRNA curves was determined by inspection of mRNA curves in every individual. The significance level was *P *< 0.05.

## Results

### Clock proteins in synovial tissue

**Figure 1:**
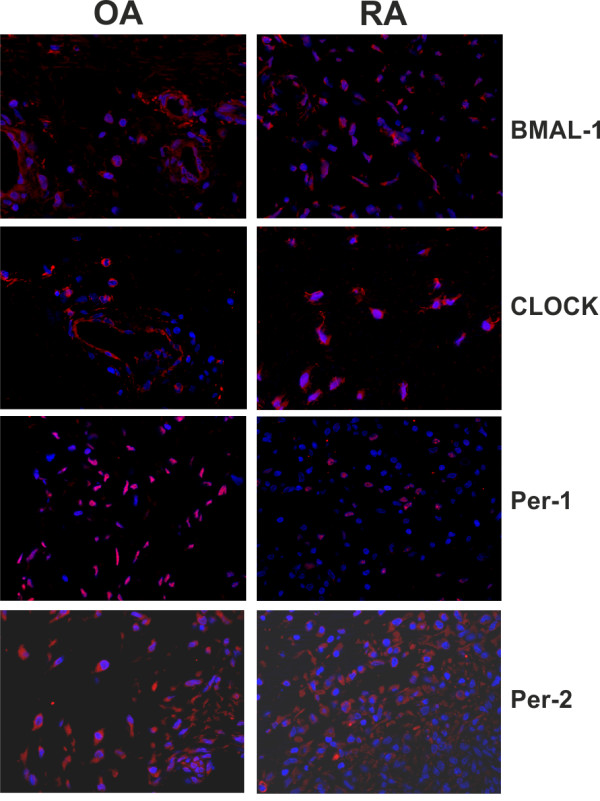
**Presence of typical clock proteins in synovial tissue**. Results of patients with osteoarthritis (OA) and rheumatoid arthritis (RA) are demonstrated. Nuclei are stained in blue with DAPI. The red staining is specific for the individual clock protein. Control staining is not given but yielded slides with negative results (only DAPI staining of nuclei is visible). Abbreviations: BMAL-1, brain and muscle arnt-like-1; DAPI, 4'-6-Diamidino-2-phenylindole; OA, osteoarthritis; Per, period homologue of Drosophila period; RA, rheumatoid arthritis.

The presence of molecular clocks is a prerequisite for endogenous cellular rhythms. This prompted us to study protein abundance in primary material of patients with OA, RA and trauma controls. Immunofluorescence demonstrated ubiquitous cellular distribution of BMAL-1, CLOCK, Period 1 and Period 2 proteins in patients with RA and OA (Figure 1). Without variation, all cells demonstrated these four clock proteins. In addition, specimens of trauma controls similarly demonstrated the four molecular clocks (data not shown).

**Figure 2:**
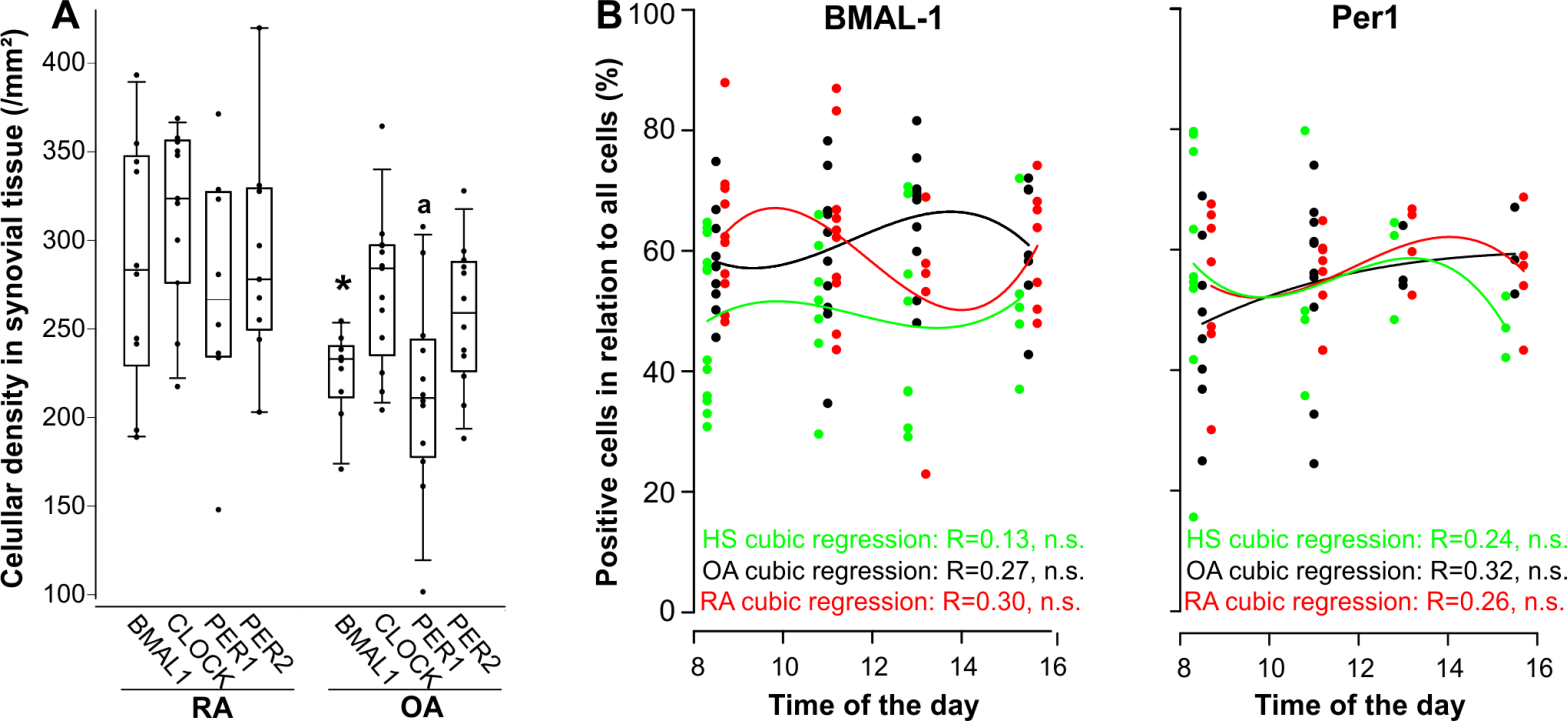
**Density of cells positive for typical clock proteins**. **A**) Density of clock protein - positive cells in synovial tissue of patients with rheumatoid arthritis (RA) and osteoarthritis (OA). *P < 0.05 vs. RA; ^a^*P *< 0.10 vs. RA. **B**) Density of cells positive for BMAL-1 (left panel) and Period 1 (Per1, right panel) over time. Synovial tissue was obtained from healthy subjects (HS, green) and patients with OA (black) and RA (red) at different time points during the day (time of operation start was grouped at 8:00 am, 11:00 am, 1:00 pm and 3:00 pm). Symbols in A) and B) indicate the mean value of cellular density of 17 randomly chosen high power fields from stained tissue of one individual patient. For example, this staining included *n *= 12 healthy subjects at time point 8:00 am. The correlation coefficient of cubic regression is given.

In order to study a possible difference between OA and RA material, cellular density of synovial cells positive for molecular clocks was investigated. While synovial density of positive cells for CLOCK and Period 2 were similar between disease groups, density of BMAL-1-positive cells was higher in RA compared to OA (Figure 2A). For Period 1-positive cells, a similar trend existed (Figure 2A). This indicates that RA synovial tissue provides an environment for increased molecular clock levels.

Since BMAL-1 and Period 1 undulate during the day, we hypothesized that this oscillation can be visible when taking patient material at different operation time points during the day. We had access to material taken between 8:00 am and 4:00 pm of a large number of patients with RA and OA as well as trauma controls. Although one would have expected a difference between morning and afternoon, no statistically significant variation of synovial density of positive cells was observed in these patient groups (Figure 2B).

In preliminary experiments, we evaluated the use of Western blots for further protein analyses of molecular clocks (data not shown). However, due to the time series with 36 time points of cell removal and lack of adequate amounts of primary cells, this technique was abandoned.

### Molecular clock mRNA in synovial fibroblasts

**Figure 3:**
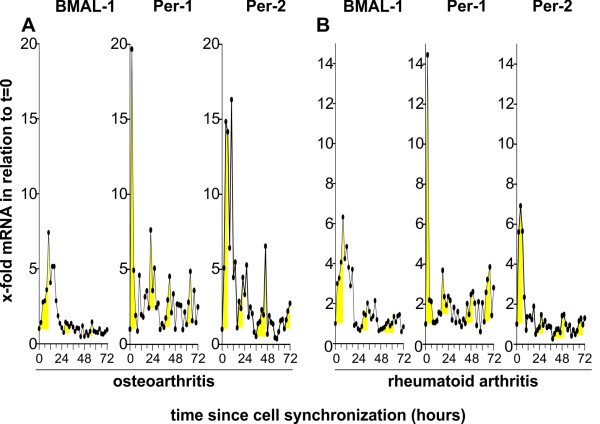
**mRNA of selected molecular clocks over time in synovial fibroblasts**. Results of patients with osteoarthritis (*n *= 6) and rheumatoid arthritis (*n *= 7) are demonstrated. One symbol represents the median value of mRNA of synovial fibroblast cultures of all included patients. The yellow area depicts the increase of the curve at different time points during an observation time of 72 hours. Typically mRNA starts with a huge peak after serum shock synchronization, which soon dies down. Abbreviations see legend to Figure 1.

Typically, quantitative PCR is used to study undulation of molecular clock mRNA over three days [15], which was also employed in the present study. After serum shock synchronization, synovial fibroblasts start with a huge rise of molecular clock mRNA as demonstrated in Figure 3. The rise of mRNA is immediate for Period 1 and Period 2 and a little delayed for BMAL-1 in both OA and RA patients (Figure 3). Using the median of mRNA of all patients in a disease group at a single time point, we were able to demonstrate oscillation curves with a total of maximal four peaks (Figure 3, yellow areas). However, time series analysis with autocorrelation did not reveal a regular pattern to determine a period of oscillation in OA and RA patients (data not shown). In other words, oscillation is masked by a low signal-to-noise ratio in OA and RA patients.

Only in fibroblasts of healthy subjects did we find a regular rhythm for BMAL-1 and Period 1 as demonstrated by autocorrelation analyses (Additional file 1, Figure S1A). These analyses revealed a period of 24 hours for both molecular clocks (Additional file 1, Figure S1A; time between red coefficients outside the confidence limits).

### Influence of IL-1β and TNF on molecular clock mRNA in synovial fibroblasts

**Figure 4:**
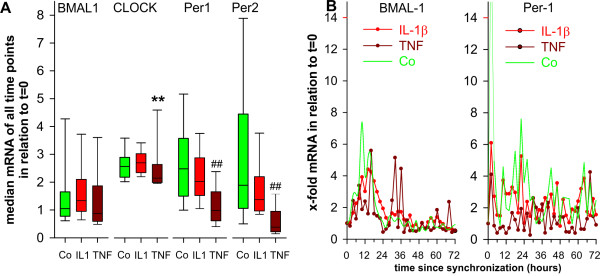
**Modulation by IL-1β and TNF of mRNA of clock proteins over time**. Results from experiments with synovial fibroblasts of patients with osteoarthritis are shown (*n *= 6). **A**) Mean of mRNA of molecular clock and influence of IL-1β (1 ng/ml) and TNF (1 ng/ml). Of every patient, the average value of mRNA was calculated from 36 time points during an observation period of 72 hours. The Box plot is generated from *n *= 6 median values of osteoarthritis patients. Box plots give the 10^th^, 25^th^, 50^th ^(median), 75^th ^and 90^th ^percentiles. **P < 0.01, ##*P *< 0.001 vs. respective control (Co). B) mRNA of selected molecular clocks over time in synovial fibroblasts of patients with osteoarthritis (*n *= 6). One symbol represents the median value of mRNA of synovial fibroblast cultures of the six patients at a given time point. The green control line is given for comparison (see Figure 3). Abbreviations see legend to Figure 1.

Since time series did not reveal molecular clock mRNA oscillation in OA and RA patients, we used the average of all 36 time points of every patient for further analyses. IL-1β and TNF are abundant proinflammatory cytokines found in patients with OA and RA. Thus, these two cytokines were studied in order to investigate the influence on molecular clock mRNA. While IL-1β had no influence on average mRNA of BMAL-1, CLOCK, Period 1 and Period 2 in OA synovial fibroblasts (Figure 4A), TNF inhibited mRNA of CLOCK, Period 1 and Period 2 (Figure 4A). This is demonstrated in the oscillation curve of Period 1 mRNA (compare green line of control conditions with brown line in Figure 4B, right panel). There are no influences on the BMAL-1 mRNA oscillation curve (Figure 4B, left panel). Similar to OA synovial fibroblasts, fibroblasts of healthy controls show a slight reduction of average CLOCK mRNA by TNF (Additional file 1, Figure S1B).

**Figure 5:**
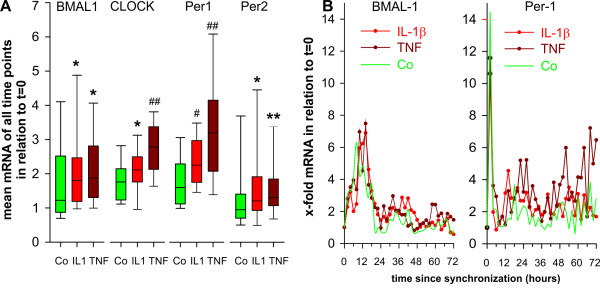
**Modulation by IL-1β and TNF of mRNA of clock proteins over time**. Results of experiments with synovial fibroblasts of patients with rheumatoid arthritis are shown (*n *= 7). A) Mean of mRNA of molecular clock and influence of IL-1β (1 ng/ml) and TNF (1 ng/ml). Of every patient, the average value of mRNA was calculated from 36 time points during an observation period of 72 hours. The Box plot is generated from *n *= 7 median values of rheumatoid arthritis patients. Box plots give the 10^th^, 25^th^, 50^th ^(median), 75^th ^and 90^th ^percentiles. *P < 0.05, **P < 0.01, #*P *< 0.005, ##*P *< 0.001 vs. respective control (Co). B) mRNA of selected molecular clocks over time in synovial fibroblasts of patients with rheumatoid arthritis (*n *= 7). One symbol represents the median value of mRNA of synovial fibroblast cultures of the seven patients at a given time point. The green control line is given for comparison (see Figure 3). Abbreviations see legend to Figure 1.

In contrast to OA synovial fibroblasts, both cytokines stimulated average mRNA of RA synovial fibroblasts (Figure 5A), which is exemplified by BMAL-1 and Period 1 mRNA oscillation curves (Figure 5B). The different protein levels demonstrated in Figure 2A supports that increased mRNA levels translates into increased protein levels.

**Figure 6:**
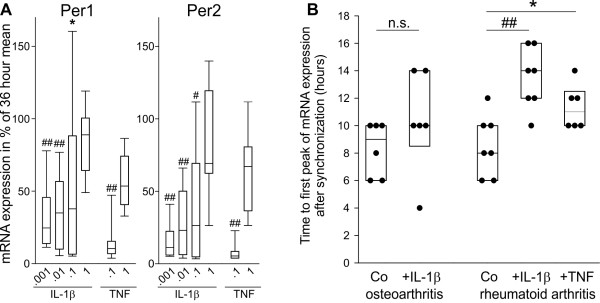
**Modulation by IL-1β and TNF of clock proteins in synovial fibroblasts**. Abbreviations see legend to Figure 1. Box plots give the 10^th^, 25^th^, 50^th ^(median), 75^th ^and 90^th ^percentiles. **A**) Mean of mRNA of Period 1 and Period 2 and dose-dependent influence of IL-1β and TNF (both ng/ml) on MH7A synovial fibroblasts. Effects on CLOCK and BMAL-1 are demonstrated in Additional file 2 Figure S2. The Box Plots are generated from at least 12 independent experiments over 36 hours (*n *= 19 for IL-1β; *n *= 12 for TNF). *P < 0.05, #*P *< 0.005, ##*P *< 0.001 vs. highest concentration. **B**) Influence of IL-1β and TNF (both 1 ng/ml) on elapsed time to first BMAL-1 mRNA peak after synchronization. Every symbol represents the time to first peak of one patient during 72 hours of observation (sample interval two hours). *P < 0.05, ##*P *< 0.001 vs. control (Co).

In order to intensify these investigations, the RA synovial fibroblast cell line MH7A was used (a large number of cells was obtained from these non-primary cells). Both, IL-1β and TNF dose-dependently increased mRNA of Period 1 and Period 2 (Figure 6A). This was similar for CLOCK mRNA but was not statistically significant for BMAL-1 mRNA (Additional file 2, Figure S2).

In order to investigate a possible cell cycle-dependent abundance of molecular clocks, the ubiquitous cell cycle progression factor Cdc2 was investigated. The oscillation curve of Cdc2 mRNA had one peak at approximately 30 hours after synchronization indicating one concerted proliferation event (Additional file 3, Figure S3A), which was largely different from the immediate peak of molecular clocks (compare Figure 3). Thus, there seems to be no direct interrelation between cell cycle and molecular clocks because no parallel or anti-parallel rise or fall of mRNA was observed. Interestingly, IL-1β and TNF increased Cdc2 mRNA in RA but not in OA or control subjects (Additional file 3, Figure S3B). In the direct comparison of RA and OA oscillation of Cdc2 mRNA, the peak was little delayed in RA compared to OA (Additional file 4, Figure S4, black letters).

### Influence of IL-1β and TNF on molecular clock mRNA oscillation characteristics

Proinflammatory cytokines might influence oscillation characteristics of molecular clocks. As mentioned above, the period of the oscillation cannot be studied due to the rapid loss of rhythmicity (see all curves in Figure 3). Thus, elapsed time from start of synchronization to the first peak of the undulation curve was scrutinized. Both, IL-1β and TNF delayed the first peak of BMAL-1 mRNA oscillation in RA synovial fibroblasts, which did not reach the significance level in OA synovial fibroblasts (Figure 6B). IL-1β and TNF did not change oscillation characteristics of other molecular clocks investigated (Additional file 4, Figure S4).

Obviously, there exist four different groups of molecular clocks as determined by the elapsed time from start to first peak (Additional file 4, Figure S4). One group has the peak between 0 and 6 h (blue), one group between 8 and 14 h (pink), another group between 14 and 17 h (red), and a fourth group between 18 and 19 h (green). Molecular clocks in these different groups reflect the different partners of the intracellular molecular clock network (Additional file 5, Figure S5). However, conditions in RA and OA synovial fibroblasts prevent one or two full oscillation cycles, which are visible in healthy fibroblasts (Additional file 1, Figure S1A).

## Discussion

Molecular clocks play an important role in coordinating circadian rhythms in the body and intracellular circadian networks in peripheral cells. It is thought that the coordinated network establishes coupling and uncoupling of pathways to support cellular function in parallel and anti-parallel fashion. To our knowledge, molecular clock proteins have not been investigated in primary synovial material of patients with OA and RA but similarly not in other rheumatic diseases.

There are three reports that linked molecular clocks to rheumatology. In mouse chondrocytes, CLOCK, Period 1 and Period 2 were found to exhibit biological rhythms after serum shock [29]. The authors found CLOCK upregulated after mechanical stress which identifies CLOCK as a possible mechanosensitive gene [29]. Another report demonstrated that melatonin inhibited Cryptochrome 1 in a mouse model of experimental arthritis [30]. However, no functional tests were performed to understand the link between melatonin, cryptochrome 1 and proinflammatory mechanisms [30].

In a third study, the authors investigated mice with collagen type II-induced arthritis using synovial and spleen cells [23]. They found marked upregulation of Period 2 independent of the light dark cycle in arthritic animals. This is somewhat similar to the upregulation observed in RA patients compared to OA (Figure 2A). In addition, they found that daily rhythm of Period 1/2 mRNA shifted six hours back and that of Bmal-1 mRNA remained constantly low indicating dampened rhythm [23]. They further demonstrated that mice deficient in Cryptochrome 1 and Cryptochrome 2 demonstrated arthritis aggravation, which might be due to upregulation of TNF [23]. They concluded that the lack of Cryptochrome gene function abrogates normal biological clock function and aggravates pathological changes in arthritis.

Our report adds more information on human subjects with OA and RA. All clock proteins are ubiquitously present in synovial cells of these patients and in trauma controls (all cells are positive). It seems that density of molecular clock-positive cells was higher in RA compared to OA, which was significant for BMAL-1. Since we have not corrected for overall cellular density, this difference might depend on increased cellular infiltration in RA compared to OA though density is relatively similar in the chronic phase of both diseases. In addition, a possible undulation of density of molecular clock-positive cells did not exist in synovial tissue. This might depend on several mechanisms, such as heterogeneous chronotypes of investigated patients (two chronotypes are known: lark and owl), different medication in the various patients that might influence molecular clock levels, short observation period from 8:00 am to 4:00 pm, and low numbers of investigated patients (type II error). Since several studies demonstrated that inflammation disturbs molecular clock oscillation in different cell types *in vivo *and *in vitro *[31-36], it might well be that typical oscillations are abrogated in inflamed tissue in OA and RA.

A closer investigation of molecular clock mRNA demonstrated that synovial fibroblasts of OA and RA patients are capable of starting the typical oscillation. However, a long-standing rhythm was not established because the signal rapidly died down. Time series analysis did not demonstrate regularity in OA and RA but in healthy fibroblasts. This can depend on the contact to the proinflammatory environment to which these cells have been adapted prior to serum shock synchronization in OA and RA [31-36].

When comparing the hardly visible oscillation in these synovial fibroblasts of OA and RA patients, fibroblasts of normal rats demonstrate excellent rhythmic oscillation [15]. From this first study in rat fibroblasts, this cell type was one of the typical peripheral cell types to study daily rhythms of the intracellular molecular clock network [37-40]. This is also true for normal human fibroblasts of the skin [40], and we also found a rhythm in fibroblasts of healthy individuals. Thus, one would have expected similar rhythms in OA and RA fibroblasts. The question appears whether or not proinflammatory factors can influence molecular clock mRNA.

Our study demonstrated that proinflammatory cytokines decreased or tended to decrease mRNA of molecular clocks in OA synovial fibroblasts. This was opposite in RA synovial fibroblast which might also explain the higher density of molecular clock - positive cells in RA synovial tissue. In these experiments, we used a dose of 1 ng/ml of IL-1β or TNF. Although this is a typical cytokine concentration to stimulate cells *in vitro*, it does not give us a dose-response effect and it is difficult to simply translate it to a situation *in vivo*. Nevertheless, these concentrations appear in the proximity of cytokine-producing cells. In order to study the subject more carefully, we applied different doses in the MH7A RA synovial fibroblast cell line. Here a clear dose-response was observable.

In addition, IL-1β and TNF both changed the time point of the first BMAL-1 mRNA peak by induction of a delay of approximately three to six hours. Similar shifts of molecular clock peaks have been demonstrated in a model of experimental arthritis [23], which indicates that inflammation can directly interfere with rhythmicity of cells. This is supported by a TNF-induced inhibition of Period 1 to 3 in NIH 3T3 fibroblasts and in mice which depends on a direct influence of BMAL-1 and CLOCK binding to the E-box [36]. In addition, expression of molecular clock genes was markedly inhibited in peripheral blood leukocytes in surgical ICU patients [41].

In further time series analyses, according to the first peak of mRNA rhythm, four different groups of molecular clocks were identified. Period 1, Period 2, Period 3, cryptochrome 1 and cryptochrome 2 (blue in Additional files 4 and 5, Figures S4 and S5) demonstrated the first peak immediately after the serum shock. BMAL-1 and CLOCK demonstrated an intermediate pattern with a peak at 8 to 17 hours (pink and red in Additional files 4 and 5, Figures S4 and S5). Furthermore, the peak of Rev-Erbα mRNA appeared late between 18 and 19 hours (green in Additional files 4 and 5, Figures S4 and S5). This sequential increase of mRNA peaks is indicative of a first successful attempt to start oscillation in these OA and RA synovial fibroblasts. However, this phenomenon is impermanent, leading to rapid destruction of the rhythm.

## Conclusions

These experiments with primary synovial tissue and fibroblasts did not demonstrate the expected oscillation of molecular clock protein or mRNA. Since proinflammatory cytokines can change the level of mRNA and time interval to peak mRNA, the proinflammatory environment most probably destroys the regular rhythm. Since synovial fibroblasts were derived from chronically inflamed tissue, molecular clock alterations are most probably programmed for a longer period of time. Disruption of coupled intracellular pathways might be a proinflammatory event.

## Abbreviations

DAPI: 4'-6-diamidino-2-phenylindole; HPA: hypothalamic-pituitary-adrenal; IL: interleukin; OA: osteoarthritis; RA: rheumatoid arthritis; SF: synovial fibroblasts; SNS: sympathetic nervous system; TNF: tumor necrosis factor.

## Competing interests

The authors declare that they have no competing interests.

## Authors' contributions

SH generated the data and draft figures, and drafted parts of the paper. RHS generated data and the final figures, and drafted the paper. Both authors gave final approval of the manuscript.

## Supplementary Material

Additional file 1 **Autocorrelation of clock factors and modulation by IL-1β and TNF of mRNA expression of clock proteins over time in synovial fibroblasts from healthy subjects**. **A**) Autocorrelation coefficient of selected clock factors. Autocorrelation is used to show a period of oscillation. mRNA expression levels of BMAL-1 and Per1 at 36 separate time points over 72 hours were used to generate autocorrelation diagrams. Every bar represents an autocorrelation coefficient generated by moving the data with the given lag number. The red lines in the graph give the 95% confidence interval of the correlation coefficients. Only red coefficients outside the red lines are significant. **B**) Mean of mRNA expression of clock factor and influence of IL-1β (1 ng/ml) and TNF (1 ng/ml). Of every person, the average value of mRNA expression was calculated from 36 time points during an observation period of 72 hours. The Box plot is generated from *n *= 4 median values of healthy subjects. Box plots give the 10^th^, 25^th^, 50^th ^(median), 75^th ^and 90^th ^percentiles. ##*P *< 0.001 vs. respective control (Co). Abbreviations see legend to Figure 1. Click here for file

Additional file 2 **Modulation by IL-1β and TNF of clock proteins in synovial fibroblasts**. Abbreviations see legend to Figure 1. Mean of mRNA expression of BMAL-1 and CLOCK and dose-dependent influence of IL-1β and TNF (both ng/ml) on MH7A synovial fibroblasts. The Box Plots are generated from at least 12 independent experiments over 36 hours (*n *= 19 for IL-1b; *n *= 12 for TNF). Box plots give the 10^th^, 25^th^, 50^th ^(median), 75^th ^and 90^th ^percentiles. *P < 0.05, **P < 0.01, #*P *< 0.005 vs. highest concentration. Click here for file

Additional file 3 **Modulation by IL-1β and TNF of mRNA expression of the cell cycle factor Cdc2 over time in synovial fibroblasts of patients with rheumatoid arthritis (RA, *n *= 7), osteoarthritis (OA, *n *= 6), and healthy subjects (HS, *n *= 4)**. Abbreviations see legend to Figure 1. **A**) mRNA expression of Cdc2 over time in fibroblasts of patients with RA and OA, and HS. The lines are generated using the median value of mRNA expression of synovial fibroblast cultures at 36 time points during 72 hours. **B**) Average of mRNA expression of clock factor and influence of IL-1β (1 ng/ml) and TNF (1 ng/ml). Of every subject, the average value of mRNA expression was calculated from 36 time points during an observation period of 72 hours. The Box plot is generated from values of patients with RA and OA, and HS. Box plots give the 10^th^, 25^th^, 50^th ^(median), 75^th ^and 90^th ^percentiles. *P < 0.05, ##*P *< 0.001 vs. respective control (Co). Click here for file

Additional file 4 **First peak after cell synchronization of individual clock factor mRNAs in OA (*n *= 6) and RA (*n *= 7) with and without additional 1 ng IL-1β/ml**. Different colors indicate co-regulated factors according to Additional file 5, Figure S5. *P < 0.05 vs. OA Cdc2+IL-1β; #*P *< 0.005 vs. OA Cdc2; ##*P *< 0.001 vs. RA BMAl-1 without IL-1β; §Cdc2 is not a clock factor but a cell cycle progression factor which indicates the cell cyle progression and, thus, proliferation. Abbreviations: BMAL-1, brain and muscle ARNT-like 1; Crypt, cryptochrome; OA, osteoarthritis; Per, period homologue of Drosophila period; RA, rheumatoid arthritis; rev-erba, V-erbA-related protein 1-related; sync, synchronization. Click here for file

Additional file 5 **Regulation of the circadian rhythm of clock proteins**. The interwoven pathways are given: 1^s^t pathway: CLOCK (C), BMAL-1(B), Period 1 to 3 (Per1, 2, 3), and Cryptochrome 1+2 (Cry1, 2); 2^nd ^pathway: Rev-Erba, BMAL-1. CLOCK and BMAL 1 dimerize and bind to E-boxes in the promotor region of Period (Per) genes, cryptochrome (Cry) genes, Rev-Erbα, and BMAL-1 activating their transcription. The corresponding proteins accumulate in the cytoplasm, translocate to the nucleus, and inhibit binding of CLOCK and BMAL-1 to the E-Boxes. This inhibition leads to a down-regulation of transcription of these genes. A very similar loop exists with respect to Rev-Erbα and BMAL-1 regulation. The degradation process via ubiquitination and SUMOylation leads to the know 24 hour cycle. Click here for file
