# Supplementary figures and images for: Disruption of rhythms of molecular clocks in primary synovial fibroblasts of patients with osteoarthritis and rheumatoid arthritis, role of IL-1β/TNF
Source: Arthritis Res Ther. 2012 May 23;14(3):R122. doi: 10.1186/ar3852 (PMC3446503; doi:10.1186/ar3852)

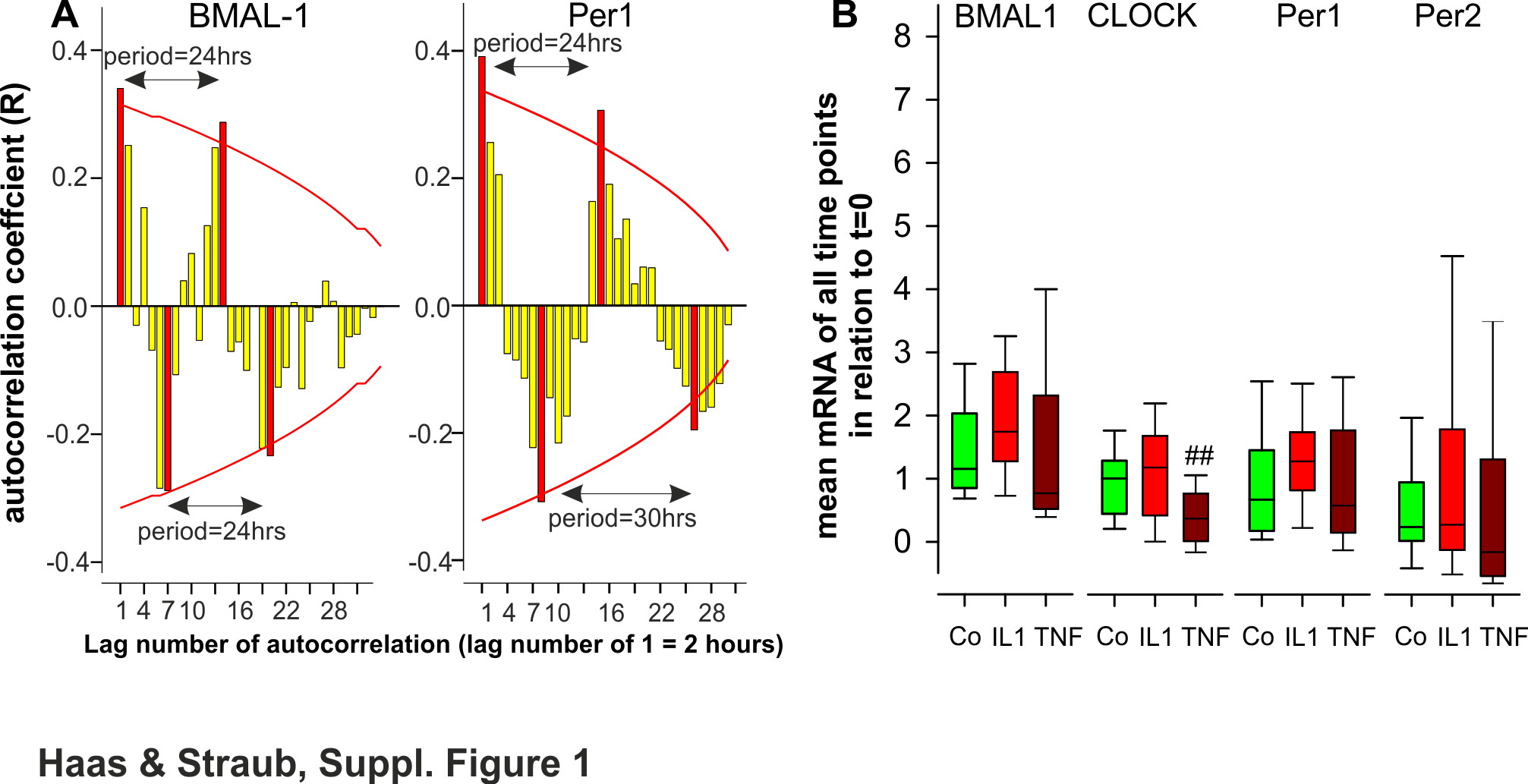

Supplement: Additional file 1 — Autocorrelation of clock factors and modulation by IL-1β and TNF of mRNA expression of clock proteins over time in synovial fibroblasts from healthy subjects. A) Autocorrelation coefficient of selected clock factors. Autocorrelation is used to show a period of oscillation. mRNA expression levels of BMAL-1 and Per1 at 36 separate time points over 72 hours were used to generate autocorrelation diagrams. Every bar represents an autocorrelation coefficient generated by moving the data with the given lag number. The red lines in the graph give the 95% confidence interval of the correlation coefficients. Only red coefficients outside the red lines are significant. B) Mean of mRNA expression of clock factor and influence of IL-1β (1 ng/ml) and TNF (1 ng/ml). Of every person, the average value of mRNA expression was calculated from 36 time points during an observation period of 72 hours. The Box plot is generated from n = 4 median values of healthy subjects. Box plots give the 10th, 25th, 50th (median), 75th and 90th percentiles. ##P < 0.001 vs. respective control (Co). Abbreviations see legend to Figure 1. [file ar3852-S1.TIFF]

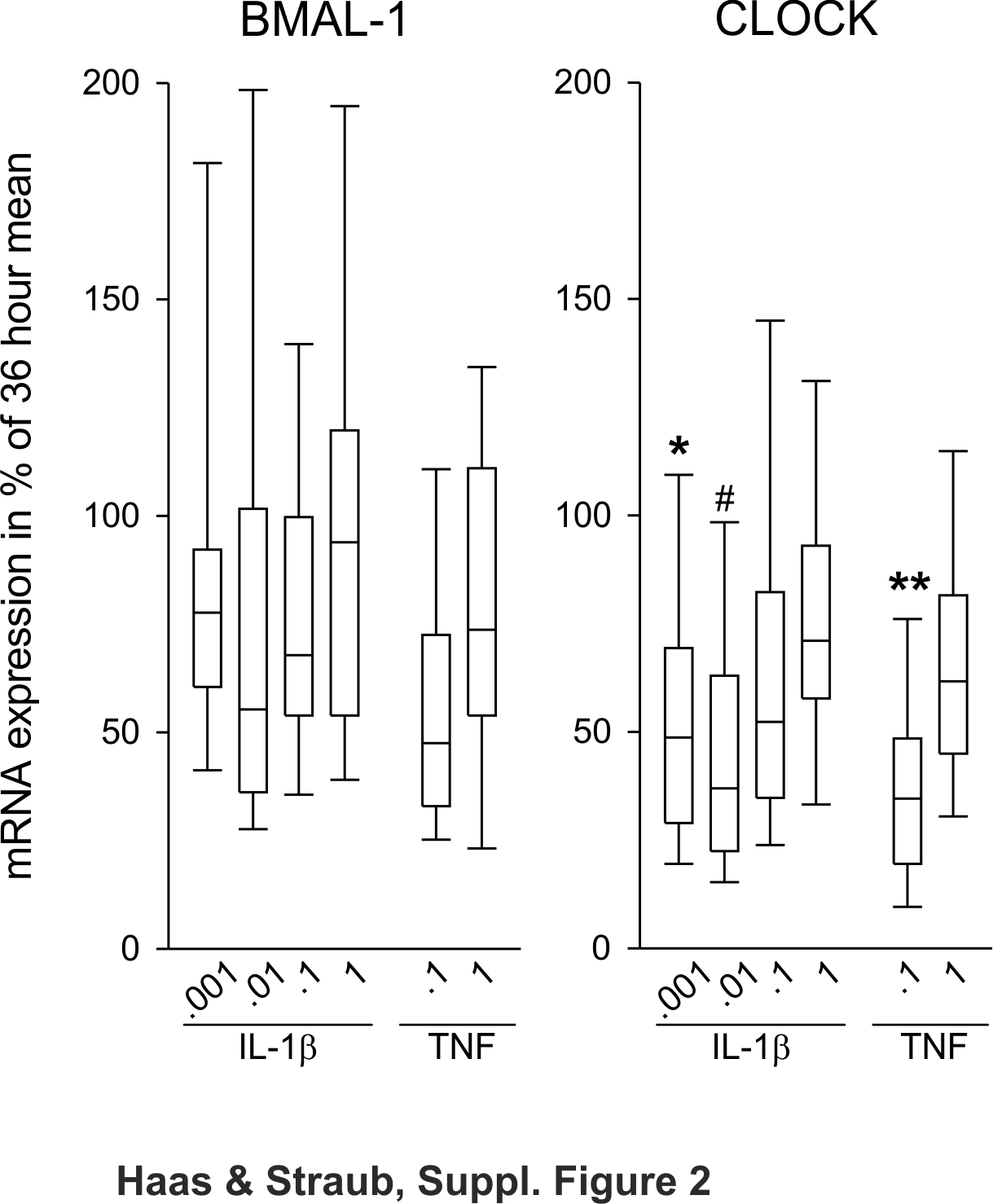

Supplement: Additional file 2 — Modulation by IL-1β and TNF of clock proteins in synovial fibroblasts. Abbreviations see legend to Figure 1. Mean of mRNA expression of BMAL-1 and CLOCK and dose-dependent influence of IL-1β and TNF (both ng/ml) on MH7A synovial fibroblasts. The Box Plots are generated from at least 12 independent experiments over 36 hours (n = 19 for IL-1b; n = 12 for TNF). Box plots give the 10th, 25th, 50th (median), 75th and 90th percentiles. *P < 0.05, **P < 0.01, #P < 0.005 vs. highest concentration. [file ar3852-S2.TIFF]

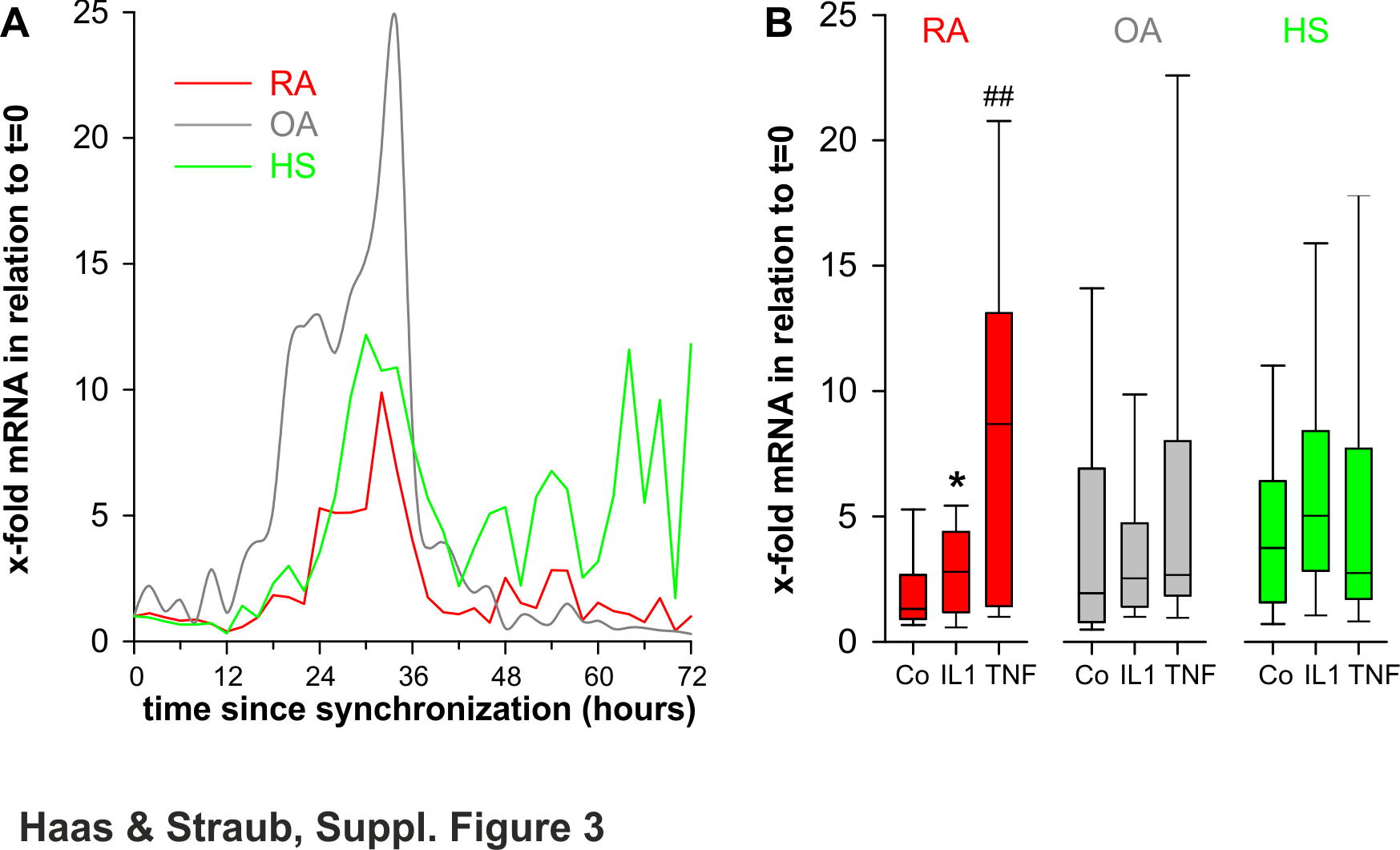

Supplement: Additional file 3 — Modulation by IL-1β and TNF of mRNA expression of the cell cycle factor Cdc2 over time in synovial fibroblasts of patients with rheumatoid arthritis (RA, n = 7), osteoarthritis (OA, n = 6), and healthy subjects (HS, n = 4). Abbreviations see legend to Figure 1. A) mRNA expression of Cdc2 over time in fibroblasts of patients with RA and OA, and HS. The lines are generated using the median value of mRNA expression of synovial fibroblast cultures at 36 time points during 72 hours. B) Average of mRNA expression of clock factor and influence of IL-1β (1 ng/ml) and TNF (1 ng/ml). Of every subject, the average value of mRNA expression was calculated from 36 time points during an observation period of 72 hours. The Box plot is generated from values of patients with RA and OA, and HS. Box plots give the 10th, 25th, 50th (median), 75th and 90th percentiles. *P < 0.05, ##P < 0.001 vs. respective control (Co). [file ar3852-S3.TIFF]

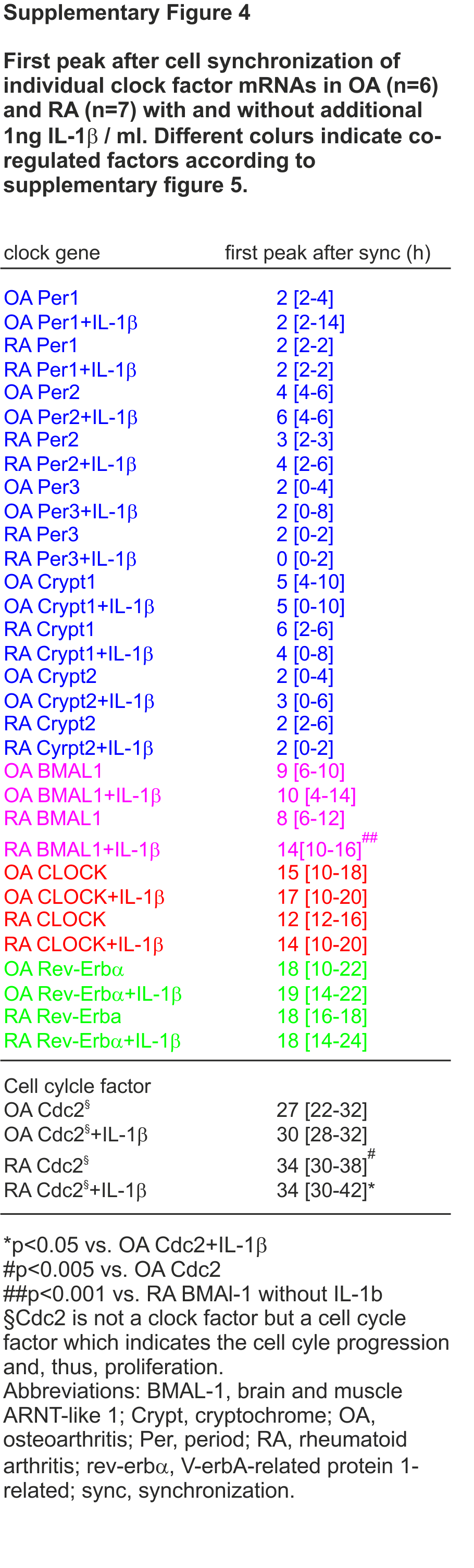

Supplement: Additional file 4 — First peak after cell synchronization of individual clock factor mRNAs in OA (n = 6) and RA (n = 7) with and without additional 1 ng IL-1β/ml. Different colors indicate co-regulated factors according to Additional file 5, Figure S5. *P < 0.05 vs. OA Cdc2+IL-1β; #P < 0.005 vs. OA Cdc2; ##P < 0.001 vs. RA BMAl-1 without IL-1β; §Cdc2 is not a clock factor but a cell cycle progression factor which indicates the cell cyle progression and, thus, proliferation. Abbreviations: BMAL-1, brain and muscle ARNT-like 1; Crypt, cryptochrome; OA, osteoarthritis; Per, period homologue of Drosophila period; RA, rheumatoid arthritis; rev-erba, V-erbA-related protein 1-related; sync, synchronization. [file ar3852-S4.TIFF]

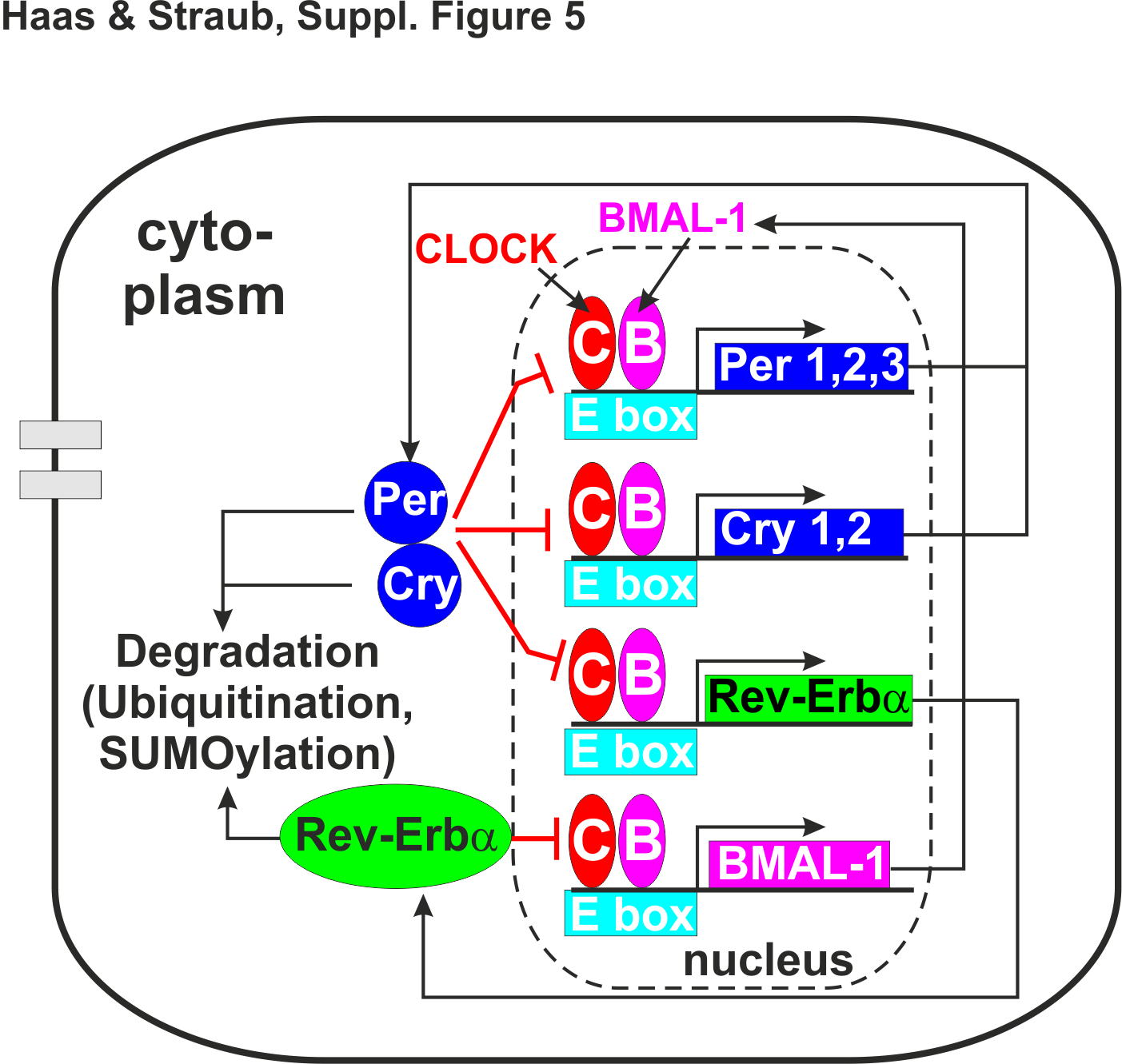

Supplement: Additional file 5 — Regulation of the circadian rhythm of clock proteins. The interwoven pathways are given: 1st pathway: CLOCK (C), BMAL-1(B), Period 1 to 3 (Per1, 2, 3), and Cryptochrome 1+2 (Cry1, 2); 2nd pathway: Rev-Erba, BMAL-1. CLOCK and BMAL 1 dimerize and bind to E-boxes in the promotor region of Period (Per) genes, cryptochrome (Cry) genes, Rev-Erbα, and BMAL-1 activating their transcription. The corresponding proteins accumulate in the cytoplasm, translocate to the nucleus, and inhibit binding of CLOCK and BMAL-1 to the E-Boxes. This inhibition leads to a down-regulation of transcription of these genes. A very similar loop exists with respect to Rev-Erbα and BMAL-1 regulation. The degradation process via ubiquitination and SUMOylation leads to the know 24 hour cycle. [file ar3852-S5.TIFF]
